# Anticholinergic burden in community-dwelling older adults: prevalence, associated factors, and agreement between measurement scales

**DOI:** 10.3389/fphar.2026.1886899

**Published:** 2026-07-31

**Authors:** Francisco De Borja Rodríguez-Salas, María Angeles Merchán-Cruz, Ricardo Ocaña-Riola, Pilar María Díez-de-Baldeón-Chicón, José Pablo Lara, Encarnación Blanco-Reina

**Affiliations:** 1 Servicio Andaluz de Salud, Health District of Málaga-Guadalhorce, Málaga, Spain; 2 Escuela Andaluza de Salud Pública, Granada, Spain; 3 IBIMA Plataforma BIONAND, Instituto de Investigación Biomédica de Málaga, Málaga, Spain; 4 Brain Health Unit, School of Medicine, Centre for Medical and Health Research (CIMES), University of Málaga, Málaga, Spain; 5 Pharmacology and Therapeutics Department, School of Medicine, University of Málaga, Málaga, Spain

**Keywords:** anticholinergic burden, anticholinergic scales, benzodiazepines, community-dwelling, medication safety, older adults, potentially inappropriate medication, primary healthcare

## Abstract

**Aim:**

Assessing cumulative anticholinergic exposure is essential in geriatric pharmacotherapy and medication safety. The present study aimed to provide real-world evidence on this issue in community-dwelling older adults. The objectives were: (i) to determine the prevalence of anticholinergic medication use; (ii) to analyze factors related to this use; and (iii) to assess the agreement between the scales applied.

**Methods:**

This multicenter cross-sectional study included 365 participants aged 65 years or older, recruited from nine urban primary healthcare centers in the city of Málaga (Andalusia, Spain), using stratified random sampling. Anticholinergic burden was calculated for all regular medications taken by participants through the application of five scales: the Anticholinergic Cognitive Burden Scale (ACB), Anticholinergic Risk Scale (ARS), Anticholinergic Drug Scale (ADS), Anticholinergic Burden Classification (ABC), and Drug Burden Index (DBI). Factors associated with the rate of anticholinergic medication use were examined using a multivariable quasi-Poisson regression model with the total number of medications included as an offset. Pairwise agreement for any anticholinergic exposure between scales was assessed using contingency tables and Cohen’s kappa coefficient.

**Results:**

Overall, 74.2% of participants were taking one or more medications with anticholinergic properties according to at least one scale. However, relevant differences were observed across the assessment instruments. Only moderate agreement was found between ADS and DBI (κ = 0.48) and between ADS and ACB (κ = 0.44); all other pairwise comparisons showed weak or very weak agreement. Anxiolytics/hypnotics (mainly benzodiazepines), antidepressants, urological antispasmodics, antihistamines, opioid analgesics, antipsychotics, and antiepileptic drugs were the therapeutic classes most frequently contributing to anticholinergic exposure. The rate of anticholinergic medication use was 30.5% higher in patients with any mental health condition, mainly anxiety and/or depression, than in those without such diagnoses (Rate Ratio = 1.305, 95% CI: 1.127–1.511).

**Conclusion:**

This study found a high prevalence of exposure to medications with anticholinergic activity among community-dwelling older adults. Although more than 50 medications were identified by the applied tools, benzodiazepines represented the therapeutic class most frequently contributing to patients’ anticholinergic burden. Clinicians should be familiar with the available tools to identify high-risk medications, consider safer alternatives, and support more appropriate prescribing.

## 1 Introduction

Acetylcholine is one of the major neurotransmitters in the human body and is extensively distributed across the central, peripheral, and autonomic nervous systems, thereby accounting for its diverse physiological actions. Consequently, drugs that block its action may produce multiple effects depending on the system and organ involved. In this context, anticholinergic burden is defined as the cumulative exposure to medications with anticholinergic properties, determined by both the number of anticholinergic drugs used and the potency of their anticholinergic effects. This burden may increase the risk of clinically relevant harms, particularly in older adults (Tune, 2001).

Anticholinergic medications may produce numerous adverse effects at both peripheral and central levels. Peripheral effects are mainly related to reduced glandular secretion and impaired smooth muscle contraction. At the central level, acute manifestations may include dizziness, sedation, confusion, and delirium (Ieong et al., 2024), whereas long-term exposure has been associated with memory impairment, cognitive decline, and even dementia (Yavuz et al., 2026; Perdixi et al., 2024; Novella et al., 2025; Zhu et al., 2025). Overall, the adverse drug reactions most frequently reported in pharmacovigilance systems include confusion, tachycardia, urinary retention, and intestinal obstruction, many of which are classified as serious (Pecquet et al., 2026). However, the most concerning adverse outcomes are probably cognitive impairment, functional decline, and mortality (Huh et al., 2024; Phutietsile et al., 2023; Poonawalla et al., 2023; Hanlon et al., 2020; Ali et al., 2020). In recent years, anticholinergic burden has also been associated with frailty and even sarcopenia (Tanaka et al., 2025). Other adverse events have also been described, including acute cardiovascular complications (Huang et al., 2023), falls (Xu et al., 2025), and an increased risk of pneumonia (Wu et al., 2023), among others.

Older adults may be particularly susceptible to these adverse effects because of reduced baseline cholinergic activity and age-related pharmacokinetic and pharmacodynamic changes (Tune, 2001). This increased vulnerability is especially relevant in the context of multimorbidity, polypharmacy, frailty, and cognitive impairment, all of which may increase the clinical impact of anticholinergic burden. Therefore, assessing cumulative anticholinergic exposure is essential in geriatric pharmacotherapy and medication safety. To this end, numerous scales have been developed, generally consisting of lists of medications classified according to their anticholinergic potential. Several systematic reviews have identified the Anticholinergic Cognitive Burden Scale (ACB), the Anticholinergic Drug Scale (ADS), the Anticholinergic Risk Scale (ARS), the Drug Burden Index (DBI), and the Anticholinergic Burden Classification (ABC) as among the most commonly used tools (Ali et al., 2020; Lisibach et al., 2021; Nishtala et al., 2016). However, none of these tools is considered a gold standard or a reference instrument in clinical practice. They differ substantially in the number of drugs included, the specific active substances considered, the weighting assigned to anticholinergic burden, and whether the prescribed dose is taken into account.

The prevalence of use of these medications varies widely. Some studies estimate that approximately half of older patients take at least one medication with anticholinergic properties (Nishtala et al., 2016). However, available data place this prevalence between 31.0% and 90.2% (Srikartika et al., 2025), while other estimates reported specifically in community-dwelling older adults vary between 6.2% and 72.8% (Pelen et al., 2025). The main factors underlying this substantial variability include methodological differences, the scales used, the healthcare setting (e.g., outpatient care, nursing homes, geriatric, psychiatric, or internal medicine units), and the geographical context. Differences between countries have been reported in several studies (Pelen et al., 2025; Cross et al., 2025) and may be partly explained by variations in prescribing patterns and national drug formularies. This supports the need for setting-specific studies, particularly in primary care. Regarding the assessment tools applied, previous research has highlighted substantial differences across scales in the estimation of anticholinergic burden (Hanlon et al., 2020; Salahudeen et al., 2015a; Tristancho-Pérez et al., 2021).

One of the first strategies to prevent medication-related harm is to quantify its magnitude across different settings and populations, as this provides the basis for targeted interventions. This may be of particular interest in Spain, a country with one of the highest life expectancies worldwide (Foreman et al., 2018). Evidence on anticholinergic burden in the Spanish population remains limited, and some previous studies have focused specifically on patients with advanced chronic conditions (Sevilla-Sánchez et al., 2018), complex chronic patients (Tristancho-Pérez et al., 2021), or individuals with cognitive impairment (López-Matons et al., 2018). The present study aimed to provide real-world evidence on this issue by combining direct patient interviews with information obtained from electronic health records. The objectives were: (i) to determine the prevalence of anticholinergic medication use in community-dwelling older adults; (ii) to analyze factors related to this use; and (iii) to assess the agreement between the scales applied.

## 2 Methods

### 2.1 Study design, setting and participants

This multicentre cross-sectional study was carried out within a primary healthcare context. All participants met the following inclusion criteria: aged 65 years or older, registered in the Spanish National Health System database and consulting in an outpatient setting (not institutionalised). The exclusion criteria were not being registered in that database, living in a nursing home, and not providing informed consent. Based on a published prevalence of patients receiving at least one medication with anticholinergic properties in primary care of 32% (Novais et al., 2025), and assuming a 5% margin of error and a 95% confidence interval, we calculated that the minimum sample size required for this study would be 334 persons, a figure that was increased by 10% to offset possible losses. Patients were recruited from nine urban primary healthcare centres in the city of Málaga (Andalusia, Spain), using stratified random sampling designed to obtain a representative sample. The study population was allocated in proportion to the size of each healthcare centre.

### 2.2 Data collection and global assessment

Study data were collected through patient interviews using a structured questionnaire. Additional information was obtained from medication packaging and electronic medical records (indication, dosage, and duration of any treatment received during the last 3 months or more). Drug exposure data included prescribed and over-the-counter treatments, as well as PRN, topical, inhaled, and herbal or plant-based products when these were part of regular use. Medications were classified into therapeutic groups according to the Anatomical Therapeutic Chemical (ATC) classification system. Polypharmacy was defined as chronic prescription of five or more drugs The questionnaire collected detailed information on regular medication use, together with clinical, functional, and sociodemographic characteristics. Clinical diagnoses were reviewed and the Charlson Comorbidity Index (CCI) was calculated. Independence in instrumental activities of daily living (IADL) was assessed with the Lawton scale. Cognitive function was evaluated using the Short Portable Mental Status Questionnaire (SPMSQ) by Pfeiffer, and mood was assessed with the 15-item Geriatric Depression Scale (GDS-15) by Yesavage. The structured questionnaire and clinical assessment instruments were administered by three physicians specialized in Family and Community Medicine, all of whom had experience in clinical practice and geriatric assessment.

### 2.3 Anticholinergic burden assessment

Anticholinergic burden was assessed for all medications received by the older adults using five anticholinergic burden tools: the Anticholinergic Cognitive Burden scale (ACB), the Anticholinergic Risk Scale (ARS), the Anticholinergic Drug Scale (ADS), the Anticholinergic Burden Classification (ABC), and the Drug Burden Index (DBI). These scales categorize drugs typically ranging from no known anticholinergic activity to high/definite anticholinergic activity. ACB, ARS and ADS scales comprised four categories (0–3): ‘0’ refers to drugs without anticholinergic effects or not listed; score ‘1’ to drugs with possible anticholinergic effects; score ‘2’ to moderate anticholinergic activity; and score ‘3’ to severe anticholinergic activity. Whereas ABC uses three categories (0, no effect; 1 mild/possible; and 2 moderate) and DBI provides a dose-dependent continuous score. The DBI is based on the formula D/(δ + D), where D represents the prescribed daily dose and δ the minimum recommended daily dose for that medication. Each individual drug contributes a value between 0 and 1, and the total DBI score for each participant was calculated by summing the individual burdens for each regular medication with anticholinergic or sedative effects. The operationalization of anticholinergic exposure for the descriptive, agreement, and regression analyses is detailed in the Statistical analysis section.

### 2.4 Statistical analysis

Anticholinergic exposure was operationalized in three complementary ways. First, participants were classified according to the burden or risk categories defined by each scale. Second, for the descriptive analysis of exposure and for pairwise agreement between scales, anticholinergic exposure was dichotomized for each tool. Participants were classified as not exposed when they had a score/category of 0 in the corresponding scale, and as exposed when they had any score/category above 0. For DBI, participants with a DBI value of 0 were classified as not exposed, whereas those with a DBI value greater than 0 were classified as exposed. Third, for the multivariable quasi-Poisson regression model, the outcome variable was the number of regular medications with anticholinergic properties received by each participant.

Exploratory data analysis and frequency tables were used to describe the study variables. To examine the relationship between the number of anticholinergic drugs and the independent variables, a multivariable quasi-Poisson regression model with semiparametric estimation by quasi-maximum likelihood was used. The total number of medications received by each participant during the study period was not homogeneous; therefore, this variable was included in the model as an offset. This allowed modelling of the rate of anticholinergic drug use, defined as the ratio between the number of anticholinergic medications and the total number of medications received during the study period. Homogeneity between primary care centres was assessed using the extended intra-class correlation coefficient for generalized linear models. Absence of multicollinearity was assessed using the Generalized Variance Inflation Factor (GVIF). Linearity of quantitative independent variables was evaluated through partial residual plots, and potential under- or overdispersion was addressed by incorporating a dispersion parameter. Statistical significance was set at α = 0.05 (Agresti, 2015). These statistical analyses were performed using R version 4.3.1 (R Foundation for Statistical Computing, Vienna, Austria), employing the R Commander graphical user interface (version 2.8–0).

Pairwise agreement for any anticholinergic use between the five scales was assessed using contingency tables and Cohen’s kappa coefficient to quantify concordances, with values ranging from −1 to 1. Kappa values were interpreted as follows: <0.20 poor agreement, 0.21–0.40 fair, 0.41–0.60 moderate, 0.61–0.80 substantial, and >0.80 almost perfect agreement.

### 2.5 Ethics

Ethics approval was obtained from the Málaga Clinical Research Ethics Committee (reference 12/2020. MSPI1). Informed consent was obtained from all patients prior to their inclusion.

## 3 Results

### 3.1 Characteristics of the study population

A total of 365 community-dwelling adults aged between 65 and 91 years were included in the study. Their mean age was 75.18 years (standard deviation 6.2), and slightly more than half were female. Overall, 24.7% of patients were aged 80 years or older. Only 18.1% lived alone; the rest lived with a partner, family member(s) or carer (professional or otherwise). The average CCI score was 3.19 (standard deviation 1.5, range 0–9). The most prevalent chronic conditions were hypertension (68.2%), dyslipidaemia (60.5%), and bone and joint disorders (mainly osteoarthritis of the knee, hip, hand and shoulder) (57.7%). Some form of psychopathology (mainly anxiety and/or depression) was present in 32.1% of the patients, and 26% suffered insomnia. According to the Lawton scale, 68.8% of the sample was independent in IADL. Only 5% of patients showed moderate-to-severe cognitive impairment according to the Pfeiffer test. Therefore, study participants had relatively preserved autonomy and cognitive status, consistent with their outpatient status. Table 1 presents selected socio-demographic, clinical and functional characteristics of the sample.

**TABLE 1:** Characteristics of the study population (N = 365).

Quantitative variables	Mean	Standard deviation
Age (years)	75.18	6.22
Lawton (IADL)	7.17	1.78
Number of comorbidities	4.36	2.37
Charlson Comorbidity Index	3.19	1.53
Number of drugs per patient	6.39	3.95

Qualitative variables	Subjects (n)	Percentage (%)
Gender Male Female	147 218	40.3 59.7
Lawton (IADL) 0-1 2-3 4-5 6-7 8	14 7 21 72 251	3.8 1.9 5.8 19.7 68.8
SPMSQ (Pfeiffer) 0-2 errors 3-4 errors 5 errors and over	317 28 18	87.3 7.7 5
GDS-15 0-5 6-9 10 and over	300 36 17	85 10.2 4.8
Specific comorbidities Hypertension Dyslipidaemia Bone and joint disorders Psychopathology Insomnia Diabetes mellitus Heart disease Respiratory disease Osteoporosis Repeated falls	249 221 210 117 95 95 71 66 58 48	68.2 60.5 57.7 32.1 26 26 19.5 18.1 15.9 13.2
Polymedication	229	62.7

IADL: Instrumental activities of daily living (ranges from 0 -low function, dependent-to 8 -high function, independent-; SPMSQ: Short Portable Mental Status Questionnaire (0–2 errors: normal mental functioning; 3-4 errors: mild cognitive impairment; 5 errors and over: moderate-severe cognitive impairment); GDS-15: Geriatric Depression Scale (0–5: no depression; 6–9: suggestive of depression; 10 and over: almost always depression).

The average number of drugs per patient was 6.39 (standard deviation 3.9; range 0–19), with a polymedication prevalence of 62.7%, being 18.9% with excessive polypharmacy. The most frequently prescribed pharmacological group according to the ATC classification was group C (cardiovascular system), as 81% of the sample chronically used at least one drug from this group, followed by groups A (alimentary tract and metabolism, 67.7%) and N (nervous system, 62.2%). The most commonly used individual medications were omeprazole, acetaminophen, simvastatin, hydrochlorothiazide (in combination), aspirin and vitamin D, followed by losartan, enalapril, and metformin.

### 3.2 Anticholinergic exposure, burden/risk categories and agreement between scales

Patients received a mean of 1.74 regular medications with anticholinergic properties (standard deviation 1.6; range: 0–8). Overall, 51 different medications were identified by at least one of the scales. These drugs belonged to different therapeutic classes, with anxiolytics/hypnotics (mainly benzodiazepines), antidepressants, urological antispasmodics, antihistamines, opioid analgesics, antipsychotics, and antiepileptic drugs being the most frequent. Representative examples included lorazepam (12.0% of patients), diazepam (8.5%), tamsulosin (7.4%), bromazepam (6.8%), tramadol (6.8%), sertraline (5.7%), and alprazolam (4.1%). Less frequently used anticholinergic medications included cetirizine (2.8%), amitriptyline (2.4%), and quetiapine (1.4%).

**FIGURE 1:**
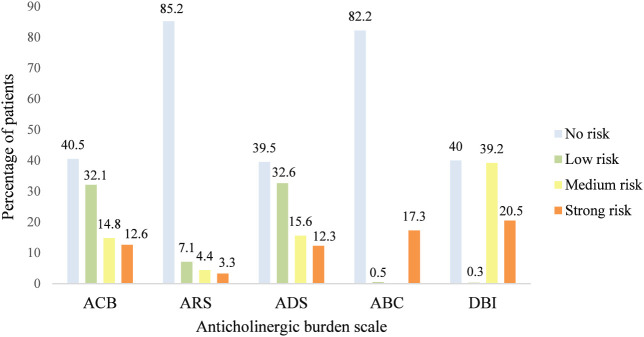
Distribution of participants according to the anticholinergic burden or risk levels defined by each tool.

Overall, 271 patients (74.2%) were taking one or more drugs with anticholinergic properties according to at least one scale. However, relevant differences were observed across the different tools. Indeed, the proportion of participants classified as not exposed according to each tool, corresponding to a score/category of 0 or a DBI value of 0, ranged from 85.2% with the ARS to 39.5% with the ADS. Using this dichotomous definition, the prevalence of any anticholinergic exposure was highest according to the ADS (60.5%), followed by the DBI (60.0%), ACB (59.5%), ABC (17.8%), and ARS (14.8%). Figure 1 provides a detailed distribution of participants across the burden or risk levels defined for each tool, taking into account that the number and meaning of categories differ across scales.

This variability was further supported by the agreement analysis based on the dichotomous classification of anticholinergic exposure for each tool. In our sample, only moderate agreement was found between ADS and DBI (κ = 0.48) and between ADS and ACB (κ = 0.44). All other pairwise comparisons showed weak or even very weak agreement, as reported in Table 2.

**TABLE 2:** Pairwise agreement between scales for dichotomous anticholinergic exposure (Cohen’s kappa and 95% confidence interval in parentheses).

Scale	ACB	ARS	ADS	ABC	DBI
ACB	1	0.17 (0.12, 0.23)	0.44 (0.35, 0.53)	0.25 (0.19, 0.31)	0.32 (0.22, 0.41)
ARS	​	1	0.14 (0.09, 0.20)	0.21 (0.08, 0.33)	0.21 (0.15, 0.26)
ADS	​	​	1	0.14 (0.08, 0.20	0.48 (0.39, 0.57)
ABC DBI	​	​	​	1	0.12 (0.06, 0.19) 1

ACB: anticholinergic cognitive burden scale; ARS: anticholinergic risk scale; ADS: anticholinergic drug scale; ABC: anticholinergic burden classification; DBI: drug burden index.

### 3.3 Factors associated with the rate of anticholinergic medication use

The variance partitioning analysis showed an extended intra-class correlation coefficient of 0.01. This value indicated that only 1% of the total variance was attributable to between-center variability. Such minimal between-center heterogeneity made it unnecessary to consider a generalized linear mixed-effects model. In the multivariate quasi-Poisson regression analysis, the rate of anticholinergic medication use was 30.5% higher in patients with psychopathological conditions (mainly anxiety and/or depression) than in those without these conditions (Rate Ratio = 1.305, 95% CI: 1.127–1.511). No statistically significant association was found between the rate of anticholinergic medication use and the other independent variables analyzed, including age, sex, hypertension, diabetes, respiratory disease, cardiovascular disease, number of comorbidities, and the Lawton and Brody Index of independence (Table 3).

**TABLE 3:** Association of independent variables with the rate of anticholinergic medication use: multivariable quasi-Poisson regression model.

Variables	Rate ratio			
	Estimate^(1)^	CI 95%^(2)^	P-value^(3)^	GVIF^(4)^
Age (years)	1.003	0.991–1.015	0.612	1.244
Gender	​	​	0.671	1.144
Male	1	​	​	​
Female	1.033	0.889–1.202	​	​
Hypertension	​	​	0.579	1.060
No	1	​	​	​
Yes	0.953	0.805–1.133	​	​
Diabetes	​	​	0.548	1.067
No	1	​	​	​
Yes	0.956	0.824–1.106	​	​
Heart Disease	​	​	0.122	1.045
No	1	​	​	​
Yes	0.879	0.745–1.032	​	​
Respiratory Disease	​	​	0.522	1.022
No	1	​	​	​
Yes	0.948	0.804–1.113	​	​
Psychopathology	​	​	<0.001	1.297
No	1	​	​	​
Yes	1.305	1.127–1.511	​	​
Number of comorbidities	0.997	0.969–1.025	0.812	1.041
Lawton (IADL)	0.975	0.942–1.011	0.167	1.311

Model parameters	Estimate	Degrees of freedom	​	​
Dispersion	0.759	​	​	​
Null deviance	301.390	344	​	​
Residual deviance	282.290	335	​	​

1 Average change in the rate of anticholinergic medication use associated with a one-unit increase in a quantitative independent variable, or the average change in the rate of anticholinergic medication use in patients from one category compared with the reference category of a qualitative independent variable.

2 95% Confidence Interval for the estimated rate ratio.

3 P-value for the estimated rate ratio.

4 Generalized variance inflation factor (GVIF) used to assess the absence of multicollinearity.

## 4 Discussion

In our study, nearly three-quarters of community-dwelling older adults were regularly taking one or more drugs with anticholinergic properties according to at least one scale, indicating a considerable overall exposure. Individual exposure also varied considerably, ranging from no such medication to up to eight per patient. Therefore, the prevalence of use of these medications was among the highest values reported in previous systematic reviews on this topic (Srikartika et al., 2025; Pelen et al., 2025). Within the European context, a recent French study reported a prevalence of 32%. However, this finding should be interpreted with caution, as that investigation did not use the same anticholinergic burden scales. Instead, prescriptions were assessed according to the list of potentially inappropriate medications included in the 2023 American Geriatrics Society Beers Criteria (B y the 2023 American Geriatrics Society Beers Criteria® Update Expert Panel, 2023) and the REMEDI [e]S tool (Roux et al., 2021), which may partly explain the lower proportion detected. At the national level, some Spanish reports have described even higher prevalence rates (76%–93%), although it should be noted that they focused on more clinically vulnerable groups, such as patients with advanced chronic conditions (Sevilla-Sánchez et al., 2018) and complex chronic patients (Tristancho-Pérez et al., 2021). Moreover, prevalence estimates in these samples also varied depending on the scale applied. Therefore, direct comparison with the literature is challenging, and these findings should be interpreted in light of methodological quality, follow-up period, the tool used to assess anticholinergic risk, participants’ age and functional status, and the care setting.

Using the dichotomous definition of exposure, we found substantial variability in detection across the five scales applied, with prevalence estimates ranging from 14.8% to 60.5%. This heterogeneity has been widely reported in the literature (Salahudeen et al., 2015a; Tristancho-Pérez et al., 2021; Pont et al., 2015; Naples et al., 2015; Mayer et al., 2017). In our sample, the ADS, DBI, and ACB yielded the highest estimates of any anticholinergic exposure, whereas the ARS identified such exposure in a much lower proportion of patients, findings that are consistent with previous studies (Salahudeen et al., 2015a; Tristancho-Pérez et al., 2021; Pont et al., 2015; Naples et al., 2015; Brombo et al., 2018). In line with this variability, weak or even very weak agreement was observed in most pairwise comparisons between scales, except for ADS-ACB and ADS-DBI, for which agreement was moderate; this has also been described by other authors (Pont et al., 2015; Naples et al., 2015). These findings once again highlight differences in drug lists, the weighting criteria assigned to each medication, whether dose is considered, and the underlying construct used to develop each tool.

Although none of the available scales is considered a gold standard, some authors have highlighted the ACB scale because of its robust development, methodological quality, and good correlations with clinical outcomes (Ali et al., 2020; Lisibach et al., 2021; Nishtala et al., 2016; D'Alia et al., 2020). It has also been described as the most frequently validated expert-based anticholinergic scale for adverse outcomes (Salahudeen et al., 2015b). Other publications have emphasized the DBI as a flexible tool, well suited for international use, associated with clinical outcomes, and particularly appropriate for longitudinal research (Mayer et al., 2017; Cardwell et al., 2015; Hilmer, 2018; Liu et al., 2024). In addition to ACB and DBI, ADS and ARS have also been recognized as high-quality scales (Lisibach et al., 2021). For the present study, we selected tools that are among the most widely used internationally and are supported by the greatest number of published validation studies (Díaz-Acedo et al., 2025). Overall, these scales may help clinicians estimate the risk associated with anticholinergic burden in their patients. However, they also have limitations, and recent evidence suggests that they may not fully capture the range of medications involved in real-world anticholinergic-related adverse events (Pecquet et al., 2026). In line with previous authors, an ideal anticholinergic burden scale should be evidence-based, internationally applicable, practical for clinical use, and capable of capturing cumulative exposure (Srikartika et al., 2025; Cardwell et al., 2015).

Benzodiazepines were the predominant medications contributing to anticholinergic exposure in our sample (mainly lorazepam, diazepam, and bromazepam, among others) and were used by approximately one-third of older adults. This finding is consistent with Novais et al., who analyzed data from a large electronic healthcare database and found that the most commonly dispensed anticholinergic or sedative medications were oxazepam, alprazolam, zopiclone, and bromazepam (Novais et al., 2025). Anxiolytics were also the drug group associated with the highest number of anticholinergic adverse drug reactions reported in the analysis of the French Pharmacovigilance Database by Pecquet et al. (Pecquet et al., 2026). Notably, the top ten countries for benzodiazepine and Z-drug consumption are European (Ma et al., 2023), with Spain standing out for the widespread use of this drug group, a pattern also observed among older adults (Lukačišinová et al., 2024). This issue has raised concern and is being addressed through several initiatives aimed at improving the rational use of these medications (Roncero et al., 2025).

Not all medications have the same anticholinergic activity and, therefore, they do not carry the same risks. Drugs such as quetiapine, olanzapine, paroxetine, carbamazepine, and amitriptyline are usually considered to have high anticholinergic potency or risk. These medications are among those most strongly associated with adverse outcomes such as cognitive impairment and frailty (Perdixi et al., 2024; Zhu et al., 2025; Tanaka et al., 2025; Cross et al., 2025). Nevertheless, among the community-dwelling older adults in our study, antipsychotic prescribing was very low, in contrast to nursing home settings, where it has been reported that one in six residents who were frail and living with cognitive impairment used a strong anticholinergic medication (Cross et al., 2025). Although most exposed patients had treatment regimens classified into low or moderate rather than high anticholinergic burden/risk categories, and despite the low use of antipsychotics, it should be noted that “light/possible” anticholinergics may be the main drivers of overall anticholinergic burden. Moreover, previous evidence suggests that high burden is more commonly accumulated through a combination of “light/possible” and “moderate/strong” anticholinergics than through “moderate/strong” anticholinergics alone (Bhatkhande et al., 2024). Therefore, when assessing prescriptions, clinicians should consider the concomitant use of several of these medications, treatment duration, and total cumulative exposure.

In the multivariable quasi-Poisson regression analysis, the rate of anticholinergic medication use was 30.5% higher in patients with any mental health condition, mainly anxiety and/or depression, than in those without such diagnoses. This association may reflect, at least in part, the previously discussed pattern of benzodiazepine use and is also supported by previous studies (Bhatkhande et al., 2024; Jun et al., 2020; Joung et al., 2019). However, given the cross-sectional design of the study, reverse causation and bidirectional relationships cannot be excluded. In our study, age, sex, number of comorbidities, and other prevalent diagnoses (hypertension, diabetes, respiratory disease, and cardiovascular disease) were not predictors of this outcome. No association was found with the Lawton and Brody Index of Independence either, although it should be noted that these older adults had an acceptable level of independence in instrumental activities of daily living. The literature shows heterogeneous and sometimes conflicting results, particularly regarding the influence of sex and age. In a recent systematic review on the prevalence and associated factors of anticholinergic medication use in community-dwelling older adults, greater anticholinergic burden was associated with female sex, lower socioeconomic status, higher comorbidity score, higher frailty probability, specific diseases, polypharmacy, and greater healthcare use, whereas increasing age was associated with both higher and lower burden (Pelen et al., 2025).

As with any study of this nature, some limitations should be acknowledged. Although we selected five scales with wide international use and accumulated validation, more recent and comprehensive anticholinergic catalogues are now available, including CRIDECO Anticholinergic Load Scale (CALS) (Ramos et al., 2022), which was specifically developed with reference to the Spanish formulary. This tool may better reflect medications currently available and prescribed in Spain, and its inclusion could have provided additional context-specific information. Future studies should consider incorporating this and other contemporary catalogues to further assess their applicability, agreement, and clinical usefulness across different healthcare settings. Another limitation is the cross-sectional design does not allow causal relationships to be established. However, it provides a snapshot of anticholinergic medication use in community-dwelling older adults and enables the identification of factors potentially related to this exposure. External validity may also be limited. Nevertheless, the multicenter nature of the study and the sampling strategy suggest that the sample may be representative of community-dwelling older adults attending urban primary healthcare centers in Málaga, and the findings should be extrapolated only to individuals with similar characteristics and care settings. In any case, such pharmacoepidemiological studies are useful for identifying and communicating medication-related risks, and may ultimately help prevent harm in clinical practice. Stratified analyses by center were not undertaken, because they were not part of the original objectives of the study. Moreover, between-center variability was negligible, as indicated by the variance-partitioning analysis, which yielded an expanded intraclass correlation coefficient of 0.01, showing that only 1% of the total variance was attributable to differences between centers.

The study also has a number of strengths. Participants were comprehensively characterized through a detailed assessment of their clinical, cognitive, and functional status, using validated instruments administered by trained family physicians. In addition, we provide real-world data on anticholinergic medication use by complementing information obtained from electronic health records with direct patient interviews. This approach may reduce the overestimation typically associated with exclusively administrative data sources, as not every medication prescribed is necessarily dispensed or taken. Additional strengths include the stratified random selection of the sample, statistical adjustment for potential confounders, and the justified selection of the anticholinergic burden scales compared in the study.

The findings of the present study contribute to the current understanding of anticholinergic burden as a relevant medication-related risk in older adults. Although this issue was underrecognized until relatively recently, growing evidence on its harmful effects has driven international efforts to minimize or rationalize the use of these medications in older populations. Indeed, these drugs, together with warnings regarding their use, have been incorporated into two of the most widely recognized international lists of potentially inappropriate medications: the Beers Criteria (By the 2023 American Geriatrics Society Beers Criteria® Update Expert Panel, 2023) and the STOPP/START criteria (Screening Tool of Older Persons’ Prescriptions/Screening Tool to Alert doctors to Right Treatment) (O'Mahony et al., 2023).

From a clinical practice perspective, the first recommendation is to periodically review the therapeutic regimen of older adults while considering their overall anticholinergic burden. When medications with such properties are prescribed, both the indication and the demonstrated therapeutic value should be carefully assessed. High-potency anticholinergic drugs should be avoided whenever possible, and alternatives with lower anticholinergic activity, dose reduction, or deprescribing should be considered. Several studies have explored initiatives aimed at reducing this exposure, including medication reviews (Gama et al., 2026) and educational interventions (Singh et al., 2024). In some cases, improvements in cognitive function have also been reported (Lundby et al., 2024; Wehran et al., 2024). However, evidence remains limited on the optimal strategies for discontinuing these treatments and on the effectiveness of interventions aimed at minimizing anticholinergic burden, underscoring the need for further research across different healthcare settings.

## 5 Conclusion

This study found a high prevalence of exposure to medications with anticholinergic properties among community-dwelling older adults, with 74.2% of participants regularly taking one or more such medications according to at least one scale. Although more than 50 medications were identified by the applied scales, benzodiazepines represented the therapeutic class most frequently contributing to patients’ anticholinergic burden. This finding highlights the need to implement proactive strategies to improve the rational use of these medications. Moreover, the rate of anticholinergic medication use was 30.5% higher in patients with any mental health condition, mainly anxiety and/or depression, than in those without such diagnoses, a result that may be related to the predominant pattern of benzodiazepine use.

Substantial differences were observed in the detection and classification of anticholinergic exposure across the five scales applied. Agreement between these tools, based on the exposed/not exposed classification, was weak or even very weak, except for ADS-ACB and ADS-DBI, for which agreement was only moderate. Therefore, the choice of scale may substantially influence the estimated prevalence of this exposure. Nevertheless, these scales may help healthcare professionals estimate the risk associated with anticholinergic use in their patients.

Assessing anticholinergic burden in the therapeutic regimens of community-dwelling older adults is clinically relevant, particularly in more vulnerable patients, such as those of advanced age or those with poorer functional or cognitive status. Clinicians should be familiar with the available tools and resources to identify high-risk medications, consider safer alternatives, and support more appropriate prescribing.

## Data Availability

The data supporting the findings of this study are not publicly available because they are being used for further research objectives and contain information that could compromise participants’ privacy. However, specific information can be obtained from the corresponding author upon reasonable request.
